# Evaluation of Universal Biomolecular Medium for Molecular Detection of Respiratory Viruses: A Paired, Multi-Platform Comparison

**DOI:** 10.3390/microorganisms14081805

**Published:** 2026-08-16

**Authors:** Martina Brandolini, Massimiliano Guerra, Giorgio Dirani, Silvia Zannoli, Alessandra Mistral De Pascali, Laura Grumiro, Laura Dionisi, Ludovica Ingletto, Giulia Gatti, Claudia Colosimo, Alessandra Scagliarini, Monica Cricca, Vittorio Sambri

**Affiliations:** 1 Unit of Microbiology, The Greater Romagna Area Hub Laboratory, 47522 Cesena, Italy; massimiliano.guerra@auslromagna.it (M.G.); giorgio.dirani@auslromagna.it (G.D.); silvia.zannoli@auslromagna.it (S.Z.); laura.grumiro@auslromagna.it (L.G.); giulia.gatti@auslromagna.it (G.G.); monica.cricca3@unibo.it (M.C.); vittorio.sambri@unibo.it (V.S.); 2 Department of Medical and Surgical Sciences (DIMEC), University of Bologna, 40138 Bologna, Italy; alessandra.depascal3@unibo.it (A.M.D.P.); laura.dionisi2@unibo.it (L.D.); ludovica.ingletto@unibo.it (L.I.); claudia.colosimo2@unibo.it (C.C.); alessand.scagliarini@unibo.it (A.S.)

**Keywords:** Universal Biomolecular Medium (UBM™), molecular diagnostics, respiratory infections, laboratory diagnosis, real-time PCR (RT-PCR)

## Abstract

Pre-analytical variables, including specimen transport media, may influence the performance of molecular assays used for respiratory pathogen detection. Universal Biomolecular Medium (UBM™) has been developed to support nucleic acid preservation and molecular testing, but evidence regarding its analytical compatibility with routinely used molecular platforms remains limited. We conducted a retrospective paired comparison of UBM™ against conventional transport and lysis media across multiple molecular platforms to evaluate the post-collection analytical compatibility of UBM. Residual respiratory specimens positive for SARS-CoV-2, influenza A virus, influenza B virus, respiratory syncytial virus, human rhinovirus, human metapneumovirus, *Haemophilus influenzae*, *Mycoplasma pneumoniae*, and *Bordetella pertussis* were analyzed. Paired aliquots generated from the same specimen were tested using the Allplex™ SARS-CoV-2/FluA/FluB/RSV Assay, Xpert^®^ Xpress CoV-2/Flu/RSV plus, Panther Fusion^®^ SARS-CoV-2/Flu A/B/RSV Assay, and selected Allplex™ respiratory panels. Analytical agreement and cycle threshold (Ct) values were compared using paired statistical analyses and Bland–Altman methods. UBM demonstrated complete qualitative concordance with comparator media across all evaluated targets and platforms, yielding 100% positive, negative and overall agreement. Although statistically significant Ct differences were observed for selected target–platform combinations, these shifts were generally small and did not affect qualitative result interpretation. Internal control performance remained stable across media. These findings support the post-collection analytical compatibility of UBM™ with the evaluated molecular workflows.

## 1. Introduction

Molecular assays represent the cornerstone of laboratory diagnosis for respiratory viral and bacterial infections [1]. The widespread implementation of real-time polymerase chain reaction (RT-PCR)-based methods has enabled rapid, sensitive, and multiplex detection of respiratory pathogens, supporting timely clinical decision-making and infection control measures [2,3,4]. While the analytical performance of molecular platforms is routinely validated, the influence of pre-analytical variables on assay outcomes is often underestimated [5,6,7].

Among pre-analytical factors, the choice of viral transport medium plays a critical role in preserving nucleic acid integrity, maintaining sample homogeneity, and ensuring compatibility with downstream extraction and amplification workflows [7,8,9,10,11]. Transport media are designed to stabilize viral particles and/or nucleic acids during sample collection, storage, and transport; however, their chemical composition may vary substantially, potentially affecting extraction efficiency, amplification kinetics, and ultimately cycle threshold (Ct) values [12,13]. Even modest Ct shifts may have implications for result interpretation, particularly in samples with low viral loads or near clinical decision threshold [14].

The unprecedented demand for molecular testing during the COVID-19 pandemic highlighted the need for alternative and flexible transport media, prompting the introduction of novel formulations intended to improve nucleic acid stabilization and biosafety [9,10,15,16,17]. Universal Transport Medium (UTM^®^) has long been used as a reference standard in respiratory virus diagnostics [11], whereas Universal Biomolecular Medium (UBM™) has been recently developed to support molecular applications by preserving nucleic acids while potentially streamlining pre-analytical workflows. Importantly, the interaction between transport media and molecular assays may be platform-dependent, as differences in extraction chemistry, amplification targets, and system integration can modulate tolerance to sample matrix variability [18].

This issue is particularly relevant in routine diagnostic laboratories, where different molecular platforms may coexist and where the same type of respiratory specimen can be processed using different pre-analytical workflows. Matrix composition, stabilizing agents, chaotropic components, and potential PCR inhibitors may differently affect nucleic acid recovery and amplification efficiency depending on the extraction system and molecular assay used. Therefore, paired-sample evaluations across multiple platforms are essential to determine whether alternative media can be safely implemented without affecting result interpretation [12].

Recent surveillance data highlight the substantial and evolving burden of respiratory viral infections worldwide and in Europe, particularly those caused by SARS-CoV-2, influenza A (FluA) and B (FluB), and respiratory syncytial virus (RSV). Integrated surveillance systems such as the European Respiratory Virus Surveillance Summary (ERVISS) [19] and international platforms including FluView [20] and WHO Global Influenza Surveillance and Response System (GISRS) [21] have documented a dynamic pattern of co-circulation and shifting predominance among these pathogens. During the 2024–2025 and 2025–2026 seasons, influenza activity in Europe increased markedly, whereas SARS-CoV-2 circulation declined, with winter positivity rates of approximately 2–3% [22]. RSV also contributed significantly to seasonal disease burden, especially among children, with overlapping epidemic waves across several EU/EEA countries [23,24,25]. In the 2025–2026 season, respiratory virus activity started earlier and intensified rapidly, with influenza test positivity reaching ~17% by mid-November 2025 and peaks near 50% [19]. The concurrent circulation of influenza, SARS-CoV-2, and RSV continues to increase healthcare pressure and highlights the importance of integrated diagnostic and surveillance approaches [26,27,28,29,30,31]. Similar global patterns reported by FluView and GISRS [20,21] confirm that seasonal respiratory viruses remain major drivers of morbidity and healthcare utilization.

Beyond SARS-CoV-2, influenza, and RSV, other viral and bacterial pathogens also contribute substantially to respiratory tract infections. Human rhinovirus (HRV) is among the most frequently detected respiratory viruses and is associated with significant morbidity across age groups [32,33], while human metapneumovirus (hMPV) is an important cause of upper and lower respiratory tract infections, particularly in pediatric, elderly, and immunocompromised populations [34,35]. In addition, bacterial pathogens such as *Haemophilus influenzae*, *Mycoplasma pneumoniae*, and *Bordetella pertussis* remain relevant causes of community-acquired respiratory infections worldwide [36,37,38], supporting the value of multiplex molecular diagnostic approaches capable of detecting a broad range of respiratory pathogens simultaneously.

While conventional viral transport media were originally developed to preserve microorganism viability, contemporary molecular diagnostics increasingly favor transport systems optimized for nucleic acid preservation and biosafety [39,40]. Conventional viral transport media are primarily designed to maintain microorganism viability and therefore require handling of potentially infectious samples throughout transport and pre-analytical processing [8]. In contrast, transport systems incorporating pathogen-inactivating reagents may improve biosafety for healthcare workers and laboratory personnel, facilitate specimen handling, and potentially simplify sample transport logistics, particularly in settings with limited access to high-containment laboratory infrastructure [40].

The relevance of this approach became particularly evident during the COVID-19 pandemic, when the need for safe and decentralized molecular testing highlighted the limitations associated with transporting infectious respiratory specimens. Richard-Greenblatt et al. demonstrated that the Copan eNAT™ transport system rapidly inactivated SARS-CoV-2 while preserving nucleic acid integrity and maintaining compatibility with molecular detection assays, supporting its use as a safer alternative to conventional transport media for diagnostic applications [41]. Similar considerations may apply to other respiratory pathogens routinely investigated by molecular methods, where preservation of nucleic acid targets rather than microorganism viability represents the primary analytical requirement [11].

UBM™ was developed as a biomolecular transport medium intended to support nucleic acid preservation while providing pathogen inactivation properties. However, before such media can be implemented in routine diagnostic workflows, it is essential to demonstrate that the inactivation chemistry does not adversely affect molecular assay performance [8,11]. Therefore, analytical comparability with established reference transport systems must be rigorously evaluated across different diagnostic platforms and respiratory targets.

In this study, we hypothesized that UBM would provide analytical performance comparable to routinely employed transport/lysis media used for the molecular detection of respiratory pathogens, without negatively affecting qualitative detection performance or qualitative result interpretation. To test this hypothesis, we performed a paired comparison of the newly developed UBM with conventional transport/lysis media applied to the molecular detection of respiratory pathogens, including SARS-CoV-2, FluA and FluB, RSV, HRV, hMPV, *H. influenzae*, *M. pneumoniae*, and *B. pertussis*. Evaluation was carried out across multiple commercially available molecular platforms. By analyzing paired samples and assessing Ct value differences using appropriate statistical methods, we aimed to characterize the post-collection analytical compatibility of UBM with downstream molecular workflows relative to currently used transport/lysis media under the tested laboratory conditions. The aim of the present study was not to evaluate specimen collection, transport preservation or biosafety characteristics of UBM, but rather to investigate whether transfer of clinical specimens into UBM after collection maintains analytical performance across commonly used molecular diagnostic platforms.

## 2. Materials and Methods

### 2.1. Study Design and Samples

This study was designed as a retrospective, paired analytical agreement study using residual clinical respiratory specimens. Universal Biomolecular Medium (UBM™, Copan Italia S.p.A, Brescia, Italy) compatibility with molecular assays for the detection of respiratory infections was evaluated. UBM is a preservation medium for nucleic acids containing Tris-EDTA, aromatic compounds and detergent. Specimens were not originally collected directly into UBM; therefore, the study did not evaluate primary specimen collection, transport and storage stability performance, or pathogen inactivation. Residual clinical respiratory specimens previously characterized for the target pathogen during routine diagnostic testing were included. For SARS-CoV-2, FluA, FluB, and RSV, 15 positive nasopharyngeal swab specimens per target, originally collected in Universal Transport Medium (UTM; Copan S.p.A., Brescia, Italy), were analyzed. UTM contains sucrose, Hanks’ Balanced Salt Solution, bovine serum albumin, buffered solution, gelatin, amino acids, antibiotics, and phenol red. Additionally, 15 samples collected in UTM and confirmed negative for SARS-CoV-2, FluA, FluB, and RSV were included to assess internal control performance. For HRV, hMPV, *H. influenzae*, *M. pneumoniae*, and *B. pertussis*, 5 positives per target and 5 shared negative nasopharyngeal aspirates collected in eSwab^®^ (Copan Italia S.p.A, Brescia, Italy) were included as exploratory targets. These analyses were considered pilot observations because the number of positive specimens was determined by sample availability and was not based on a formal sample size calculation for demonstrating analytical equivalence. eSwab consists of a modified Liquid Amies medium containing sodium chloride, potassium chloride, calcium chloride, magnesium chloride, monopotassium phosphate, disodium phosphate, sodium thioglycolate, and distilled water. Positive samples were selected to include a range of viral loads based on Ct values obtained at initial clinical testing, in order to reduce spectrum bias.

For all targets, sample selection was based on the availability of eligible residual clinical specimens collected during the study period; therefore, the study cohort was convenience-based rather than determined by a formal a priori sample size calculation. The resulting sample size was considered adequate for the paired exploratory comparison performed in this study, but was not intended to provide sufficient statistical power to formally demonstrate analytical equivalence for each individual pathogen or target.

Eligible specimens were required to have a documented prior clinical result, known specimen type, to be fresh (collected within the prior 24 h and maintained at 2–8 °C after testing for clinical purposes) or stored at ≤−70 °C, no more than one previous freeze–thaw cycle, with sufficient residual volume to prepare paired test conditions. Samples with undocumented storage conditions, more than one freeze–thaw cycle, insufficient residual volume, visible leakage or gross contamination, visually compromised appearance, or unresolved conflicting prior results were excluded. All samples were anonymized and handled in accordance with institutional policies for the use of leftover diagnostic material.

### 2.2. Molecular Assays

Molecular testing was performed using commercially available platforms according to the manufacturers’ instructions.

For SARS-Cov-2, FluA, FluB and RSV, the following platforms were included in the study:

- Allplex™ SARS-CoV-2/FluA/FluB/RSV Assay [42] (Seegene, Seoul, Republic of Korea), targeting S (spike), RdRP (RNA-dependent RNA polymerase) and N (nucleocapsid) genes of SARS-CoV-2, M (matrix) gene of FluA, NS2 (non-structural) gene of FluB and M gene of RSV;
- Xpert^®^ Xpress CoV-2/Flu/RSV plus [43] (Cepheid, Sunnyvale, CA, USA), targeting N, E (envelope) and RdRP genes of SARS-CoV-2, M, PB2 (basic polymerase) and PA (acidic protein) of FluA, M and NS genes of FluB, and N gene of RSV;
- Panther Fusion^®^ SARS-CoV-2/Flu A/B/RSV Assay [44] (Hologic, Marlborough, MA, USA), targeting ORF1ab (open reading frame) gene of SARS-CoV-2, M gene of FluA and FluB and N gene of RSV.

While the three SARS-CoV-2 targets included in the Allplex SARS-CoV-2/FluA/FluB/RSV Assay generate three independent amplification signals, each corresponding to a distinct Ct value that was analyzed separately, a different interpretation applies to the FluA1 and FluA2 targets in the Xpert Xpress CoV-2/Flu/RSV plus.

For HRV, hMPV, *H. influenzae*, *M. pneumoniae* and *B. pertussis*, the following diagnostic assays were employed:

- Allplex™ Respiratory Panel 2 (Seegene, Seoul, Republic of Korea), for the detection of Human metapneumovirus (MPV);
- Allplex™ Respiratory Panel 3 (Seegene, Seoul, Republic of Korea), for the detection of Human rhinovirus A/B/C (HRV);
- Allplex™ PneumoBacter Assay (Seegene, Seoul, Republic of Korea), for the detection of *H. influenzae*, *M. pneumoniae* and *B. pertussis*.

These multiplex assays are capable of detecting a broader range of respiratory viral and bacterial pathogens than those evaluated in the present study. However, only hMPV, HRV, *H. influenzae*, *M. pneumoniae* and *B. pertussis* were included in the exploratory analyses reported here, while the remaining detectable targets were outside the scope of the study and were not systematically assessed.

All runs included assay-specific internal controls to monitor extraction efficiency, amplification performance, and inhibition; kit controls were performed according to the relevant instructions for use.

### 2.3. Sample Preparation

Frozen residual specimens were thawed at 2–8 °C until fully thawed, gently mixed by five inversions, and processed after verification of pre-analytical eligibility. For each specimen, paired aliquots were generated from the same original sample to ensure direct comparability between transport conditions.

**Figure 1:**
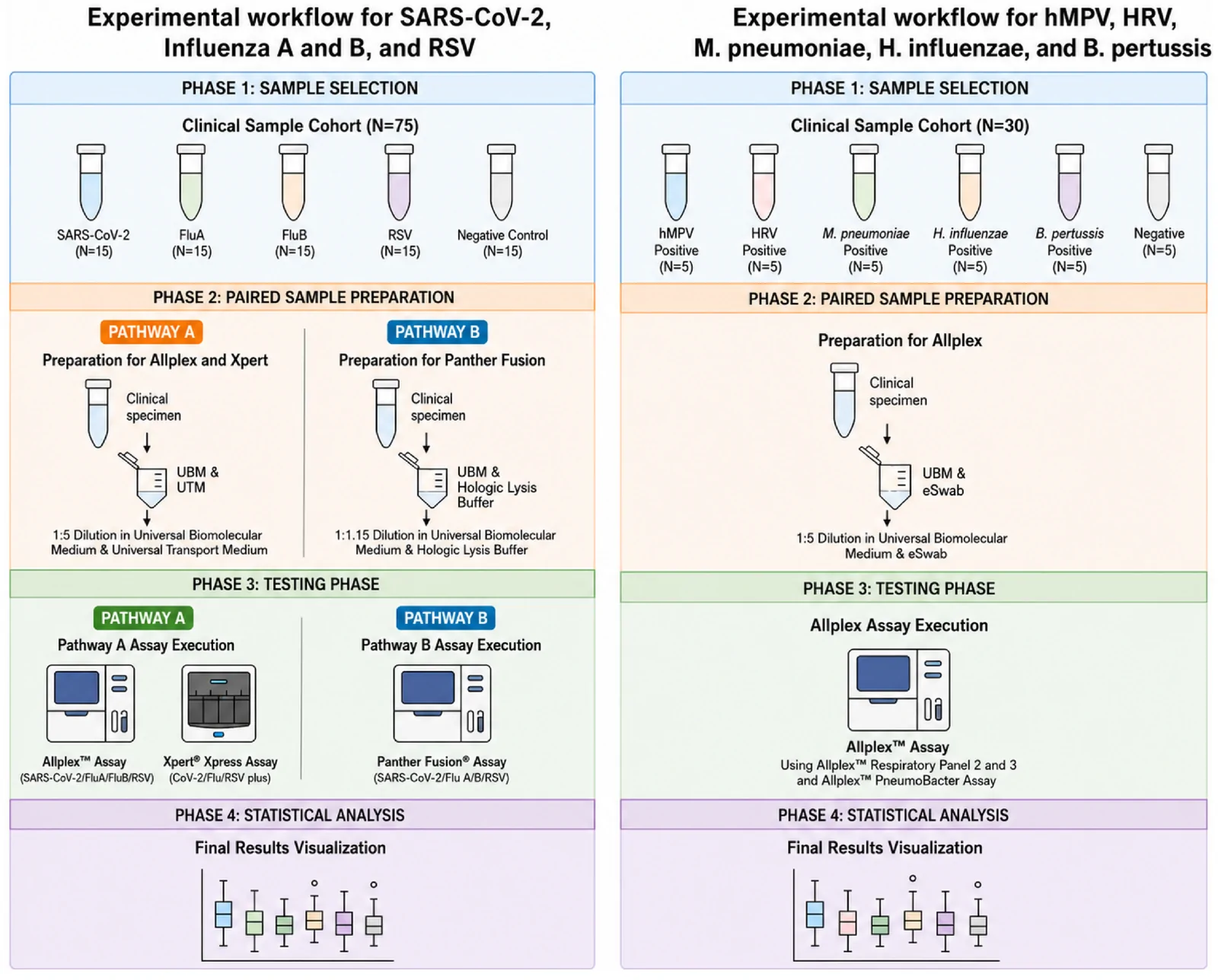
Experimental workflows for the comparative evaluation of respiratory and bacterial molecular assays. The schematic illustrates the experimental workflows used for the analysis of clinical specimens. The left panel describes the workflow for SARS-CoV-2, influenza A and B viruses, and respiratory syncytial virus (RSV), including sample selection, paired sample preparation using Universal Biomolecular Medium (UBM) and the respective comparator media, testing with Allplex™, Xpert^®^ Xpress, and Panther Fusion^®^ assays, and subsequent statistical analysis. The right panel shows the workflow for human metapneumovirus (hMPV), human rhinovirus (HRV), *Mycoplasma pneumoniae*, *Haemophilus influenzae*, and *Bordetella pertussis*, comprising 30 clinical specimens (five positive specimens for each target and five negative controls). Samples were prepared in parallel using UBM and eSwab and subsequently analyzed using the Allplex™ Respiratory Panel 2 and 3 and Allplex™ PneumoBacter Assay. For both workflows, the final phase comprised comparative statistical analysis and graphical visualization of the assay results. The figure was created with the assistance of Google NotebookLM (Google LLC, Mountain View, CA, USA, web-based version) and subsequently reviewed and edited by the authors for scientific accuracy.

For SARS-CoV-2, FluA, FluB and RSV detection through molecular assays performed on Seegene Allplex and Xpert Xpress platforms, positive samples were diluted 1:5 (1 part specimen and 4 parts medium) in UTM and UBM (500 μL of target-positive or target-negative UTM in 2 mL of UTM and UBM). For Panther Fusion assays, samples diluted 1:1.5 in UBM (704 μL of target-positive or target-negative UTM in 1 mL of UBM) were compared with samples processed using the manufacturer-recommended Panther Fusion™ Specimen Lysis Tubes buffer as the reference comparator, in accordance with the system’s standard workflow (500 μL of target-positive or target-negative UTM in 710 μL of Hologic proprietary lysis buffer). The dilution ratio was intentionally chosen to match the specimen-to-buffer proportion recommended by the manufacturer for processing respiratory specimens in the proprietary Panther Fusion™ Specimen Lysis Tubes buffer, thus maintaining comparability with the standard Panther Fusion pre-analytical workflow. For HRV, hMPV, *H. influenzae*, *M. pneumoniae* and *B. pertussis* samples were diluted 1:5 (1 part specimen and 4 parts medium) in eSwab and UBM. Following transfer, paired aliquots were mixed by pulsed vortexing and incubated for 10 min at room temperature before analysis. When feasible, paired conditions were tested in the same analytical run, in blinded and randomized order, to minimize inter-run variability. Negative specimens were processed using the same paired preparation scheme. Sample preparation is depicted in Figure 1.

### 2.4. Data Analysis and Statistics

Ct values obtained from paired aliquots of the same original specimen were recorded for each target–platform combination. For each comparison, Ct values obtained after transfer into UBM were compared with those obtained under the corresponding comparator transport or lysis condition. Qualitative agreement was assessed separately for each target by calculating positive percent agreement, negative percent agreement, overall percent agreement, and discordance from 2 × 2 contingency tables. The n/N values represent the number of concordant positive or negative results relative to the corresponding reference totals. The 95% confidence intervals for all proportions were calculated using the Wilson score method. Ct values were summarized as mean ± standard deviation, together with the mean paired Ct difference between conditions.

The distribution of paired Ct differences was assessed using the Shapiro–Wilk test. When the distribution of paired differences was compatible with normality, conditions were compared using the paired Student’s *t*-test. When normality was not met, the Wilcoxon signed-rank test was applied. A two-sided *p* value < 0.05 was considered statistically significant. For each target–platform combination, the mean bias was defined as the average paired Ct difference calculated as comparator minus UBM. The mean bias and its 95% confidence interval were calculated together with the lower and upper 95% limits of agreement and their corresponding 95% confidence intervals.

The 95% confidence interval for the mean bias was calculated using the t distribution. Confidence intervals for the limits of agreement were calculated using the parametric Bland–Altman method, accounting for uncertainty in both the mean difference and its standard deviation. A priori, a mean Ct bias within ±3.0 Ct was defined as a pragmatic criterion for the magnitude of the mean analytical difference because it approximates a one-log10 difference in target concentration and remains within the range of inter-assay variability reported for molecular diagnostic platforms [45,46].

This threshold was not considered sufficient, either alone or in combination with qualitative concordance, to establish equivalence or interchangeability. Agreement was interpreted by considering the mean bias and its precision, the limits of agreement and their confidence intervals, and the distribution of individual paired differences. Results exceeding the prespecified ±3.0 Ct mean-bias threshold were considered not to meet this criterion, irrespective of statistical significance in paired comparisons. Paired significance tests were used to assess evidence of systematic differences between conditions; a non-significant result was not interpreted as evidence of equivalence or interchangeability.

Qualitative agreement, including concordant positive, concordant negative, and discordant results, was reviewed descriptively when applicable. Internal control performance was also evaluated descriptively to assess possible inhibition or extraction/amplification failure. Statistical analyses were performed using R (version-4.6.1).

## 3. Results

### 3.1. SARS-CoV-2, FluA, FluB and RSV

With regard to SARS-CoV-2, FluA and B and RSV, a total of 75 residual nasopharyngeal swab specimens were included in the study, comprising 15 positive samples for each viral target evaluated and 15 shared negative samples. Paired aliquots generated from the same original specimen were analyzed under the two comparator conditions for each platform.

For all targets, the detection performance of the assays reached 100% positive and negative agreement, as well as 100% overall agreement and 0% discordance for all analyzed targets (Table 1).

Given this complete concordance in qualitative results, subsequent analyses focused on the comparison of Ct values obtained from diluted samples processed in the two transport media, in order to assess potential differences in analytical performance. Results are summarized in Table 2.

To complement the paired statistical comparisons, Bland–Altman analysis was used to characterize the magnitude and specimen-level variability of the Ct differences observed between UBM and the corresponding comparator condition. Mean bias estimates, their 95% confidence intervals, and the lower and upper limits of agreement with their corresponding confidence intervals are reported in Table 3. These estimates were interpreted descriptively and were not considered, either alone or in combination with qualitative concordance, sufficient to demonstrate equivalence or interchangeability.

#### 3.1.1. Allplex SARS-CoV-2/FluA/FluB/RSV Assay

For the Allplex SARS-CoV-2/FluA/FluB/RSV Assay, paired analysis showed statistically significant differences between UBM and UTM for selected targets. Ct values for the SARS-CoV-2 N gene were significantly higher in UBM than in UTM (24.9 ± 5.9 vs. 21.4 ± 5.5; t(14) = 5.8, *p* < 0.001). A statistically significant difference was also observed for the RdRp gene, again with higher Ct values in UBM than in UTM (23.1 ± 3.5 vs. 21.0 ± 4.2; t(14) = 3.5, *p* = 0.004). By contrast, the S gene did not show a statistically significant difference between media (19.6 ± 3.0 in UBM vs. 20.4 ± 3.9 in UTM; t(14) = 1.5, *p* = 0.163). For FluA, the assay showed a small but statistically significant difference between transport media, with slightly higher Ct values in UBM than in UTM (25.7 ± 2.9 vs. 24.9 ± 2.3; t(14) = 2.2, *p* = 0.043). No statistically significant systematic difference was detected for FluB (24.0 ± 2.6 in UBM vs. 24.0 ± 3.0 in UTM; t(14) = 0.1, *p* = 0.906). For RSV, the Wilcoxon signed-rank test did not detect a statistically significant systematic difference between UBM and UTM (21.9 ± 4.9 vs. 22.3 ± 5.0; *p* = 0.164). In negative specimens, no statistically significant systematic difference was detected for the exogenous internal control (19.9 ± 0.4 in UBM vs. 19.9 ± 0.6 in UTM; t(14) = 0.09, *p* = 0.931) or for the endogenous internal control (26.3 ± 2.1 in UBM vs. 26.2 ± 1.8 in UTM; t(14) = 0.1, *p* = 0.886). These non-significant findings should not be interpreted as evidence of equivalence.

**Figure 2:**
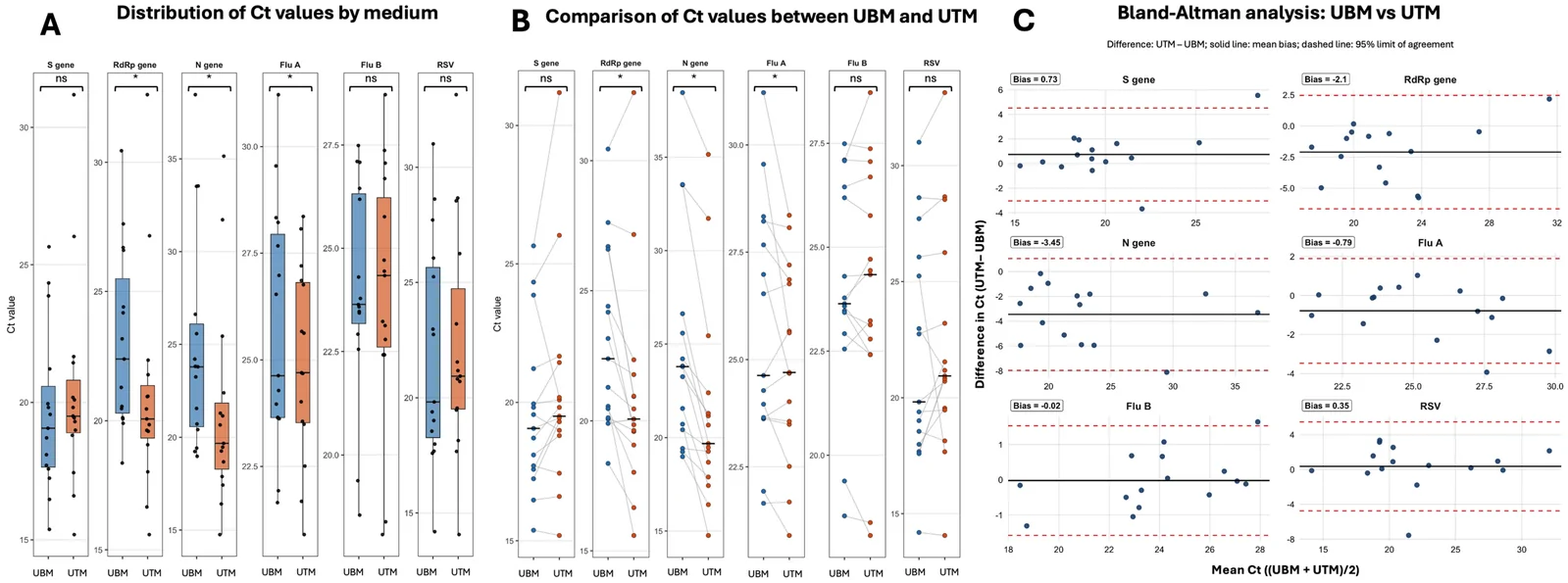
Distribution, paired comparison, and agreement analysis of Ct values obtained with the Seegene Allplex SARS-CoV-2/FluA/FluB/RSV Assay comparing UBM and UTM. (**A**) Box-and-whisker plots showing the distribution of cycle threshold (Ct) values for SARS-CoV-2 targets, including S, RdRp, and N genes, as well as Influenza A, Influenza B, and RSV, in samples processed in Universal Biomolecular Medium (UBM) and Universal Transport Medium (UTM). Central lines represent medians, boxes indicate interquartile ranges, whiskers show data dispersion, and individual observations are overlaid. (**B**) Paired comparison plots of Ct values between UBM and UTM for each target. Individual paired measurements are connected by line segments, illustrating within-sample variation attributable to the transport medium. Horizontal black bars denote mean Ct values. (**C**) Bland–Altman plots assessing agreement between UBM and UTM. The difference in Ct values, calculated as UTM − UBM, is plotted against the mean Ct for each pair, calculated as (UBM + UTM)/2. The solid line represents the mean bias, and dashed lines indicate the 95% limits of agreement. ns = non-statistically significant, * = statistically significant.

Bland–Altman analysis was used to further evaluate agreement between UBM and UTM across all evaluated targets. The corresponding mean-bias estimates, confidence intervals, and limits of agreement are reported in Table 3. Differences were calculated as UTM − UBM; therefore, negative bias values indicate higher Ct values in UBM, whereas positive bias values indicate higher Ct values in UTM. For SARS-CoV-2 targets, the S gene showed a small positive bias (+0.73 Ct; 95% LoA, −3.05 to +4.52), indicating slightly higher Ct values in UTM. Conversely, the RdRp gene showed a negative bias (−2.10 Ct; 95% LoA, −6.66 to +2.46). The Allplex SARS-CoV-2 N target showed a mean bias of −3.45 Ct (95% CI, −4.72 to −2.18), which exceeded the prespecified ±3.0 Ct threshold and therefore did not meet the predefined criterion for acceptable mean analytical difference. The corresponding limits of agreement ranged from −7.95 Ct (95% CI, −10.18 to −5.73) to +1.05 Ct (95% CI, −1.17 to +3.28), indicating substantial specimen-level variability. Therefore, the Allplex SARS-CoV-2 N results should be interpreted as showing a systematic and variable Ct difference between UBM and UTM. This finding was consistent with the paired statistical analysis and suggests a target-specific effect of the transport medium on SARS-CoV-2 N and RdRp gene detection using the Allplex platform. For FluA, a modest negative bias was observed (−0.79 Ct; 95% LoA, −3.47 to +1.90), consistent with slightly higher Ct values in UBM. FluB showed an almost negligible mean bias (−0.02 Ct; 95% LoA, −1.58 to +1.53), indicating limited mean systematic deviation within the evaluated specimen set. RSV displayed a small positive bias (+0.35 Ct; 95% LoA, −4.76 to +5.47), indicating slightly higher Ct values in UTM on average, although the wide limits of agreement indicate substantial specimen-level variability. Across all targets, no clear proportional bias was observed over the Ct range, suggesting that differences between media were not strongly dependent on viral load within the tested Ct interval. Overall, the Allplex results showed target-dependent agreement between UBM and UTM. While several targets showed relatively small mean differences, the SARS-CoV-2 N target exceeded the prespecified ±3.0 Ct mean-bias criterion and showed wide limits of agreement. Therefore, the Allplex findings do not support a conclusion of interchangeability across all evaluated targets, and the observed target-specific variability should be taken into account when interpreting Ct values. The observed increase in Ct values with UBM may be more critical in low-viral-load specimens, where amplification occurs close to the assay detection threshold, and small Ct shifts could potentially result in discordant qualitative classification between transport media. Results for the Allplex SARS-CoV-2/FluA/FluB/RSV Assay are reported in Figure 2.

#### 3.1.2. Xpert Xpress CoV-2/Flu/RSV Plus

Among the platforms tested, Xpert Xpress CoV-2/Flu/RSV plus showed high qualitative concordance between UBM and UTM. For SARS-CoV-2, paired *t*-test analysis showed no statistically significant difference in Ct values between the two media (21.3 ± 3.4 in UBM vs. 21.4 ± 3.2 in UTM; t(14) = 0.5, *p* = 0.626). For FluA, two distinct amplification signals were analyzed. No statistically significant systematic difference was detected for either signal. For FluA1, the Wilcoxon signed-rank test showed no statistically significant difference between conditions (22.9 ± 3.2 in UBM vs. 22.5 ± 2.8 in UTM; *p* = 0.607). Similarly, for FluA2, the Wilcoxon signed-rank test did not detect a statistically significant systematic difference between conditions (24.4 ± 3.7 in UBM vs. 24.0 ± 3.4 in UTM; *p* = 0.532). For FluB, a statistically significant difference was observed, with slightly lower Ct values in UBM than in UTM (22.1 ± 2.6 vs. 22.7 ± 2.9; t(14) = 2.9, *p* = 0.012). However, the absolute difference was small and did not alter qualitative classification in the evaluated specimens. RSV Ct values were not significantly different between conditions (22.7 ± 4.2 in UBM vs. 22.9 ± 4.2 in UTM; t(14) = 0.7, *p* = 0.492). Analysis of the assay internal control in negative samples also showed no statistically significant difference between UBM and UTM (28.4 ± 0.2 vs. 28.3 ± 0.3; t(14) = 1.1, *p* = 0.301). The non-significant paired comparisons were not interpreted as evidence of equivalence.

**Figure 3:**
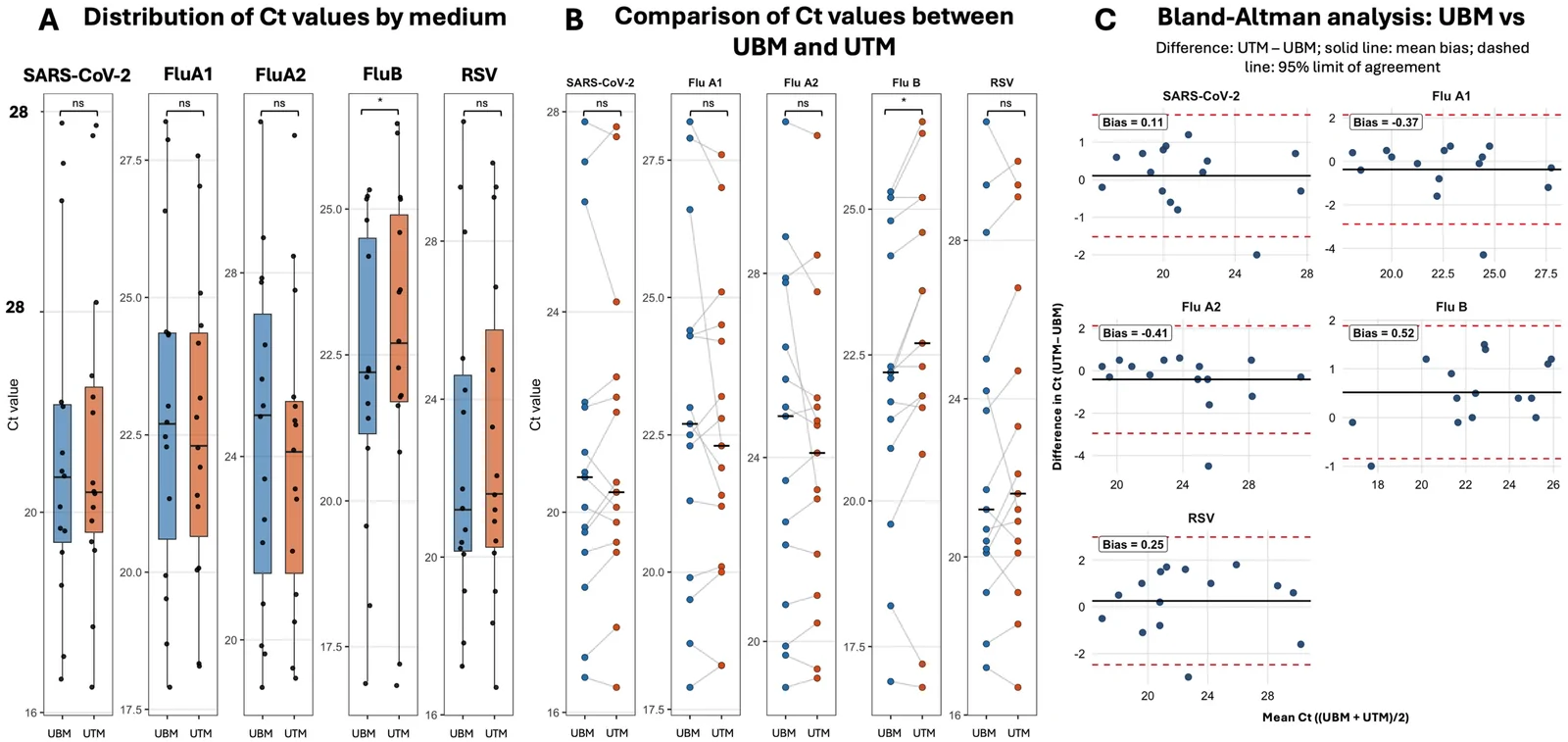
Distribution, paired comparison, and agreement analysis of Ct values obtained with the Xpert Xpress CoV-2/Flu/RSV plus platform comparing UBM and UTM. (**A**) Box-and-whisker plots showing the distribution of cycle threshold (Ct) values for SARS-CoV-2, Influenza A (FluA1 and FluA2), Influenza B, and RSV in paired nasopharyngeal specimens processed in Universal Biomolecular Medium (UBM) and Universal Transport Medium (UTM). (**B**) Paired Ct comparison between UBM and UTM for each target. Each line connects measurements derived from the same specimen, highlighting within-sample variability attributable to transport medium. (**C**) Bland–Altman plots illustrating agreement between UBM and UTM. The difference in Ct values, calculated as UTM − UBM, is plotted against the mean Ct [(UBM + UTM)/2], with mean bias shown as the solid line and 95% limits of agreement shown as dashed lines. ns = non-statistically significant, * = statistically significant.

Bland–Altman analysis was used to further assess agreement between UBM and UTM on the Xpert Xpress CoV-2/Flu/RSV plus platform. The corresponding mean-bias estimates, confidence intervals, and limits of agreement are reported in Table 3. Differences were calculated as UTM − UBM; therefore, positive bias values indicate higher Ct values in UTM, whereas negative bias values indicate higher Ct values in UBM. For SARS-CoV-2, the mean bias was close to zero (+0.11 Ct; 95% LoA, −1.52 to +1.73), indicating limited mean systematic deviation between conditions. For FluA1 and FluA2, small negative biases were observed (−0.37 Ct; 95% LoA, −2.89 to +2.14, and −0.41 Ct; 95% LoA, −2.95 to +2.12, respectively), consistent with slightly higher Ct values in UBM than in UTM on average. However, the magnitude of these mean differences was limited, and no clear proportional trend was observed across the evaluated Ct range. For FluB, Bland–Altman analysis showed a positive bias (+0.52 Ct; 95% LoA, −0.85 to +1.89), consistent with slightly lower Ct values in UBM than in UTM. This finding was consistent with the paired statistical comparison, in which FluB was the only Xpert Xpress target showing a statistically significant difference between conditions. For RSV, the mean bias was small and positive (+0.25 Ct; 95% LoA, −2.47 to +2.98), consistent with minimally lower Ct values in UBM than in UTM on average. Overall, the observed mean biases were small within the evaluated specimen set. Nevertheless, these estimates and their limits of agreement describe the observed agreement under the tested conditions and do not establish equivalence or interchangeability between UBM and UTM. Results for Xpert Xpress CoV-2/Flu/RSV plus are reported in Figure 3.

#### 3.1.3. Panther Fusion SARS-CoV-2/Flu A/B/RSV Assay

For the Panther Fusion SARS-CoV-2/Flu A/B/RSV Assay, the comparator condition consisted of the manufacturer-provided Panther Fusion Specimen Lysis Tubes buffer, against which UBM was evaluated. For SARS-CoV-2, a statistically significant difference was observed, with slightly higher mean Ct values in UBM than in the Panther Fusion Specimen Lysis Tubes buffer comparator (19.9 ± 3.4 vs. 19.6 ± 3.5; t(14) = 2.3, *p* = 0.040). Although statistically significant, the absolute Ct shift was small. For FluA, no statistically significant systematic difference was detected between UBM and the comparator condition (24.4 ± 3.4 in UBM vs. 24.4 ± 3.4 in Panther Fusion Specimen Lysis Tubes buffer; Wilcoxon signed-rank test, *p* = 0.589). Likewise, no statistically significant systematic difference was detected for FluB (25.9 ± 2.8 in UBM vs. 25.9 ± 3.3 in Panther Fusion Specimen Lysis Tubes buffer; t(14) = 0.1, *p* = 0.900). For RSV, a statistically significant difference was observed, with higher mean Ct values in UBM than in the Panther Fusion Specimen Lysis Tubes buffer comparator (23.3 ± 3.9 vs. 22.3 ± 3.2; t(14) = 2.9, *p* = 0.011). The observed difference did not alter qualitative classification in the evaluated specimens. Internal control analysis in negative samples revealed a small but statistically significant difference, with lower Ct values in UBM than in the comparator condition (33.4 ± 0.5 vs. 34.1 ± 1.1; t(14) = 2.2, *p* = 0.041). The non-significant paired comparisons for FluA and FluB were not interpreted as evidence of equivalence.

**Figure 4:**
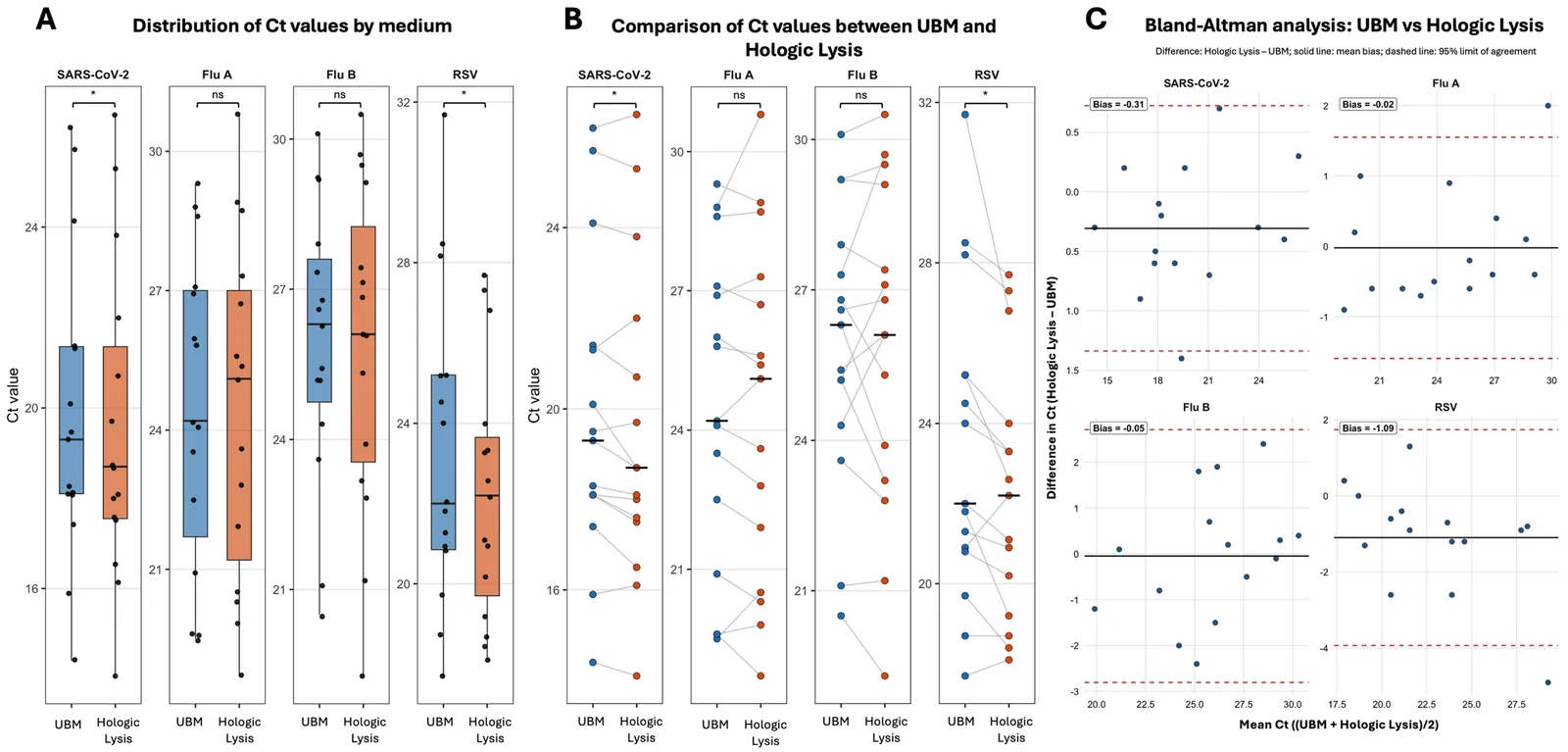
Distribution, paired comparison, and agreement analysis of Ct values obtained with the Panther Fusion SARS-CoV-2/Flu A/B/RSV Assay comparing UBM and Panther Fusion Specimen Lysis Tubes buffer. (**A**) Box-and-whisker plots showing the distribution of cycle threshold (Ct) values for SARS-CoV-2, FluA, FluB, and RSV in paired nasopharyngeal specimens processed in Universal Biomolecular Medium (UBM) and the manufacturer-recommended Panther Fusion Specimen Lysis Tubes buffer. (**B**) Paired Ct comparison between UBM and Panther Fusion Specimen Lysis Tubes buffer for each viral target. Individual paired observations are connected to illustrate within-sample variation. (**C**) Bland–Altman plots assessing agreement between UBM and Panther Fusion Specimen Lysis Tubes buffer. The difference in Ct values, calculated as Panther Fusion Specimen Lysis Tubes buffer − UBM, is plotted against the mean Ct [(UBM + Panther Fusion Specimen Lysis Tubes buffer)/2], with mean bias shown as the solid line and 95% limits of agreement shown as dashed lines. ns = non-statistically significant, * = statistically significant.

Bland–Altman analysis was performed to further assess agreement between UBM and the Panther Fusion Specimen Lysis Tubes buffer on the Panther Fusion platform. The corresponding mean-bias estimates, confidence intervals, and limits of agreement are reported in Table 3. Differences were calculated as Panther Fusion Specimen Lysis Tubes buffer − UBM; therefore, negative bias values indicate higher Ct values in UBM, whereas positive bias values indicate higher Ct values in the Panther Fusion Specimen Lysis Tubes buffer. The mean bias was −0.31 Ct (95% LoA, −1.34 to +0.72) for SARS-CoV-2, −0.02 Ct (95% LoA, −1.59 to +1.55) for FluA, −0.05 Ct (95% LoA, −2.80 to +2.71) for FluB, and −1.09 Ct (95% LoA, −3.93 to +1.74) for RSV. These results indicate small mean systematic differences for FluA and FluB, while SARS-CoV-2 and particularly RSV showed higher Ct values in UBM on average. This pattern was consistent with the paired statistical analysis, in which SARS-CoV-2 and RSV showed statistically significant Ct differences between conditions. Across targets, no clear proportional trend was observed over the evaluated Ct range. Overall, the Bland–Altman estimates describe target-specific agreement within the evaluated specimen set. Although no qualitative discordances were observed, these findings do not establish equivalence or interchangeability between UBM and the manufacturer-provided lysis buffer. Results are presented in Figure 4.

### 3.2. hMPV, HRV, H. influenzae, M. pneumoniae and B. pertussis

Within this limited exploratory specimen set, paired qualitative results were concordant between UBM and eSwab across all analyzed targets. For each target, positive percent agreement, negative percent agreement, and overall percent agreement were 100%, with 0% discordance, indicating complete qualitative agreement within the evaluated specimen set. However, because only five positive specimens were available for each pathogen, these results describe the observations in this limited dataset and should not be interpreted as demonstrating equivalent analytical performance between the two processing conditions (Table 4).

Given the limited number of samples available for each target, no additional quantitative assessment of Ct value differences was undertaken, warranting further investigation.

## 4. Discussion

In this paired, multi-platform comparison, post-collection transfer into UBM was associated with preserved qualitative molecular detection within the evaluated specimen set, while quantitative Ct differences were target- and platform-dependent. Importantly, the scope of these findings is restricted to the analytical compatibility of UBM following transfer of previously collected clinical specimens and should not be interpreted as a validation of UBM as a primary specimen collection or transport medium. Because all specimens were originally collected in UTM or eSwab and subsequently aliquoted into UBM and comparator media, the present study did not assess direct specimen collection into UBM, collection efficiency, nucleic acid stability during transport or storage, preservation over defined time and temperature intervals, or other pre-analytical factors that may influence molecular detection. Results indicate that, within the tested experimental conditions, transfer of clinical specimens into UBM did not result in discordant qualitative molecular results on the evaluated platforms.

The most relevant finding from the analytical comparison was that the quantitative differences observed between UBM and the comparator media were not accompanied by changes in qualitative classification. Across all three platforms evaluated, no discordant qualitative results were observed: all positive specimens remained positive, and all negative specimens remained negative under paired comparison conditions. This finding indicates that, within the analytical range represented by the selected sample set, post-collection transfer into UBM did not alter the qualitative classification of the evaluated specimens. However, it should not be interpreted as evidence that UBM would necessarily preserve qualitative performance under all pre-analytical or clinical conditions. Although statistically significant Ct shifts were identified for selected target–platform combinations, these shifts did not alter qualitative interpretation or final result assignment in the specimens examined.

This distinction is particularly important when interpreting the potential relevance of Ct differences. Statistical significance does not necessarily equate to biological or clinical relevance. In paired analyses with relatively controlled experimental conditions, even modest and analytically consistent Ct differences may achieve statistical significance, especially when variability within pairs is low. However, the practical meaning of these shifts depends on their magnitude, reproducibility, direction, and position relative to assay decision thresholds. In the present study, the magnitude of the mean Ct differences was target- and platform-dependent. Although several mean biases were limited to a fraction of a cycle or approximately 1 Ct, the Allplex SARS-CoV-2 N target showed a mean bias of −3.45 Ct and therefore exceeded the prespecified ±3.0 Ct criterion. Nevertheless, the absence of discordant results in the present sample set does not establish that such shifts would be clinically irrelevant in specimens with target concentrations close to an assay-specific decision threshold. This is particularly relevant for low-positive specimens, in which relatively small analytical differences may potentially influence qualitative classification.

The observed quantitative differences were platform- and target-dependent. Xpert Xpress generally showed relatively small mean biases, Panther Fusion showed target-specific differences, and Seegene Allplex exhibited a more heterogeneous pattern. The magnitude and variability of Ct differences therefore varied across platform–target combinations. Importantly, the Allplex SARS-CoV-2 N target exceeded the prespecified ±3.0 Ct threshold for mean bias and showed wider limits of agreement, indicating greater specimen-level variability. The width of the limits of agreement reflects the observed specimen-level variability, while the corresponding confidence intervals quantify the uncertainty surrounding the mean bias and limits-of-agreement estimates (Table 3). These findings further support a cautious, platform- and target-specific interpretation of post-collection analytical agreement rather than a general conclusion of interchangeability. On Xpert Xpress, the high qualitative agreement observed between UBM and UTM across the evaluated respiratory targets provides evidence of post-collection analytical compatibility under the conditions tested. However, these findings do not establish interchangeability of the two media for all specimen types, collection procedures, transport conditions, or laboratory workflows. Any potential use of UBM on this platform would require independent verification under the specific conditions in which it is intended to be used.

On Panther Fusion, qualitative classification was preserved within the evaluated specimen set, while quantitative agreement was target-dependent. In this system, UBM was compared with the Panther Fusion Specimen Lysis Tubes buffer rather than UTM, reflecting the actual pre-analytical workflow recommended by the manufacturer. Statistically significant systematic differences were detected for SARS-CoV-2, RSV, and the internal control, whereas no statistically significant systematic differences were detected for Influenza A or Influenza B. The non-significant comparisons should not be interpreted as evidence of equivalence. Importantly, however, the magnitude of the mean differences remained limited, and no discordant qualitative results were observed. These findings indicate that, in the experimental comparison performed, specimens transferred into UBM retained the same qualitative classification as specimens processed using the manufacturer-provided lysis buffer within the evaluated specimen set. However, this comparison should not be interpreted as validation of UBM as a substitute for the proprietary lysis buffer in the complete Panther Fusion workflow.

For the Panther Fusion platform, paired aliquots derived from specimens originally collected in UTM were processed either after transfer into UBM or using the manufacturer-provided Panther Fusion Specimen Lysis Tubes buffer. Therefore, this comparison was limited to post-collection sample processing under the specific laboratory conditions evaluated. Although no qualitative discordances were observed, the experiment did not assess direct primary specimen collection into UBM and does not demonstrate that UBM can replace the proprietary lysis buffer within the complete manufacturer-recommended workflow. Collection efficiency, nucleic acid preservation during transport or storage, stability under defined time and temperature conditions, pathogen inactivation, and other pre-analytical variables were not evaluated and require dedicated prospective validation.

The Seegene Allplex assay showed a more heterogeneous pattern, with statistically significant systematic differences detected for two SARS-CoV-2 targets and for FluA, while no statistically significant systematic differences were detected for the other evaluated targets or internal controls. These non-significant comparisons were not interpreted as evidence of equivalence. These findings indicate that UBM may exert target-dependent effects on Ct values in assays relying on multiplex amplification of several genomic regions. Such behavior is not unexpected. Multiplex assays are inherently more sensitive to subtle changes in sample matrix composition, extraction efficiency, or amplification kinetics because multiple primer/probe sets compete within the same reaction environment. The fact that not all targets moved in the same direction or to the same extent suggests that the interaction between transport medium and assay performance is not simply a generalized inhibition or enhancement phenomenon, but rather a more complex platform- and target-specific effect. Importantly, the Allplex SARS-CoV-2 N target did not meet the prespecified ±3.0 Ct mean-bias criterion. The mean bias was −3.45 Ct (95% CI, −4.72 to −2.18), and the limits of agreement ranged from −7.95 Ct (95% CI, −10.18 to −5.73) to +1.05 Ct (95% CI, −1.17 to +3.28). These estimates indicate both a systematic difference and substantial specimen-level variability and do not support interchangeability between UBM and UTM for this target. Accordingly, the observed variability further supports the need to consider UBM compatibility on a platform- and assay-specific basis rather than extrapolating the findings obtained with one molecular system to other diagnostic workflows.

The interpretation of Ct variability remains a key aspect in evaluating the impact of transport media. Ct values are not absolute measurements of viral burden in the strict sense; rather, they are assay-dependent semi-quantitative readouts influenced by specimen quality, collection technique, transport conditions, extraction efficiency, target design, amplification chemistry, and instrument-specific analysis algorithms. In the present study, the observed Ct differences should therefore be interpreted as analytical differences between paired sample-processing conditions rather than as evidence of differences in viral burden or clinical significance.

The absence of discordant qualitative calls should not lead to the conclusion that Ct shifts are universally irrelevant. Their importance increases as results approach the lower limit of detection or the assay-specific cutoff. In low viral load specimens, where amplification may occur late and close to the positivity threshold, a shift of even 1 Ct could, in principle, influence whether a sample is classified as detected, presumptive, or not detected. The present study did not demonstrate such an effect, but the limited representation of near-threshold specimens prevents its exclusion. Additional validation in cohorts enriched for near-cutoff samples would therefore be valuable to better define the potential impact of UBM-related Ct differences.

A further consideration is that the present study was not designed or statistically powered to evaluate UBM performance according to target load or under conditions approaching the analytical limit of detection. The distribution of Ct values was limited to the range represented in the study cohort, and the number of specimens available in the higher-Ct subgroups was insufficient to support robust comparisons. Consequently, the absence of qualitative discordance observed in the present dataset should not be extrapolated to weakly positive specimens or samples with target concentrations close to the assay-specific LoD, given the limited and convenience-based sample size. Dedicated studies incorporating a larger number of near-threshold specimens and replicate testing around the LoD will be required to characterize this aspect more reliably.

Another important aspect concerns result reporting. In most clinical microbiology laboratories, molecular respiratory virus results are reported qualitatively, whereas Ct values, if available, are either not released or are interpreted with caution. In the present study, the preserved categorical agreement observed after transfer into UBM indicates that the change in sample-processing condition did not alter qualitative result assignment within the evaluated specimen set. However, this observation should not be interpreted as evidence supporting clinical reportability, changes in laboratory reporting practices, or operational flexibility. Because Ct values may be affected by pre-analytical conditions, the observed differences should be considered when comparing results generated under different sample-processing conditions. Further studies would be required to determine whether any observed Ct differences have implications for clinical interpretation or reporting in specific diagnostic workflows. In laboratories that include Ct values in the laboratory information system or communicate them to clinicians upon request, it would be advisable to recognize that a change in sample-processing condition may introduce target- and platform-specific systematic differences relative to historical data generated with other media. This does not imply that Ct values become unreliable, but rather that cross-comparison of absolute Ct numbers obtained under different pre-analytical conditions should be avoided or interpreted prudently.

Although the primary focus of this study was on SARS-CoV-2, influenza viruses, and RSV, a limited exploratory analysis regarding additional respiratory pathogens, including hMPV, HRV, *H. influenzae*, *M. pneumoniae*, and *B. pertussis*, was also performed. For each of these targets, only five positive specimens were available for paired testing, substantially limiting the robustness of any analytical inference. The observed Ct differences were therefore considered descriptive observations only and were not interpreted as evidence of equivalence or suitability of UBM for these additional pathogens. Given the convenience-based sample selection and the limited number of positive specimens available for these pathogens (n = 5 per target), these results should be regarded as preliminary and hypothesis-generating. Larger studies with adequate numbers of positive specimens would be required to determine whether the observed patterns are reproducible.

From a translational perspective, the present findings provide preliminary analytical evidence for further investigation rather than evidence sufficient to support routine clinical implementation of UBM. In particular, the study does not establish whether UBM can be used for direct primary specimen collection, whether it can replace conventional transport media throughout the entire pre-analytical pathway, or whether it can replace manufacturer-specific lysis reagents within complete sample-to-answer workflows. These questions require dedicated prospective validation studies designed around the intended clinical workflow.

Several limitations should be acknowledged when interpreting the findings. First, the study used a finite number of specimens per target, and although the paired design increases sensitivity for detecting matrix-related effects, the sample size may not capture the full biological variability encountered in routine diagnostics. An important limitation of the present study is that the sample size was convenience-based and determined by the availability of eligible residual clinical specimens rather than by a formal a priori sample size calculation. Consequently, the study was not designed or powered to formally demonstrate analytical equivalence for each individual pathogen or target. This limitation is particularly relevant for all considered targets. These analyses should therefore be regarded as pilot observations that provide preliminary analytical information rather than definitive evidence of equivalence. Larger, adequately powered studies will be required to confirm these findings [8,9,11,12]. Second, the study focused on nasopharyngeal swabs and on a selected set of respiratory viral assays; performance may differ in other specimen types, on other platforms, or with pathogens not examined here. Third, while no discordant calls were observed, the sample panel may have included relatively few specimens lying exactly at the limit of detection, which is the setting in which Ct shifts would be most likely to influence qualitative classification. Fourth, and critically, specimens were not collected directly into UBM. Instead, aliquots from specimens initially collected in routine media were transferred into UBM before molecular testing. Consequently, the present study specifically evaluates post-collection analytical compatibility and does not address specimen collection efficiency, nucleic acid preservation during transport, storage stability, pathogen inactivation, or other pre-analytical variables. Finally, the comparator condition differed by platform, with Panther Fusion compared against the Panther Fusion Specimen Lysis Tubes buffer rather than UTM, reflecting the experimental workflow but limiting strict cross-platform comparability of the pre-analytical contrast.

Overall, post-collection transfer of respiratory specimens into UBM was associated with preserved qualitative molecular detection within the evaluated platforms and specimen set, despite target- and platform-dependent Ct differences. These findings characterize target- and platform-specific post-collection analytical agreement under the laboratory conditions investigated, but do not establish analytical equivalence, universal interchangeability with the comparator conditions, suitability of UBM as a primary specimen collection or transport medium, or replacement of manufacturer-specific lysis reagents. The limited and convenience-based sample set, including the limited representation of near-threshold specimens, further restricts the extent to which these findings can be generalized to routine clinical populations. Future prospective and adequately powered studies should evaluate direct specimen collection into UBM, standardized transport and storage conditions, specimen stability, a broader range of clinical matrices and pathogens, and a greater representation of low-positive and near-threshold specimens before conclusions regarding routine clinical implementation can be drawn.

## 5. Conclusions

In this multi-platform paired comparison study, post-collection transfer into UBM was associated with preserved qualitative molecular detection within the evaluated specimen set. Quantitative agreement was target- and platform-dependent. In particular, the Allplex SARS-CoV-2 N target showed a mean bias of −3.45 Ct (95% CI, −4.72 to −2.18), exceeding the prespecified ±3.0 Ct criterion, with limits of agreement ranging from −7.95 to +1.05 Ct and indicating substantial specimen-level variability. Therefore, the findings do not support universal interchangeability between UBM and the comparator conditions. Rather, they characterize target- and platform-specific post-collection analytical agreement under the tested laboratory conditions.

The preserved qualitative agreement observed in the evaluated specimen set should not be interpreted as evidence of clinical equivalence or as support for routine implementation. In particular, the limited representation of low-positive and near-threshold specimens prevents exclusion of potential effects of Ct differences on qualitative classification close to assay-specific decision thresholds. Dedicated prospective and adequately powered studies will therefore be required to determine whether these findings can be extended to direct specimen collection into UBM, different pre-analytical conditions, additional specimen types, a greater representation of low-positive and near-threshold specimens, and routine clinical workflows.

## Figures and Tables

**Table 1:** Analytical performance of UBM compared with UTM for the detection of SARS-CoV-2, FluA, FluB and RSV. Results are reported for each tested assay and each target organism. Positive % agreement, negative % agreement, overall % agreement and discordance % are reported for each target organism. Numerators and denominators (*n*/*N*) indicate the number of concordant results over the total number of expected positive or negative samples. Ninety-five percent confidence intervals (95% CI) are shown in brackets. Confidence intervals for proportions were calculated using the Wilson score method. Percentages are reported using the decimal separator applied throughout the manuscript.

Assay	Target	Positive % Agreement [*n*/*N*; 95% CI]	Negative % Agreement [*n*/*N*; 95% CI]	Overall % Agreement [*n*/*N*; 95% CI]	Discordance % [*n*/*N*; 95% CI]
**Allplex SARS-CoV-2/FluA/FluB/RSV Assay**	**SARS-CoV-2**	100.0% (15/15; 79.6–100.0%)	100.0% (60/60; 94.0–100.0%)	100.0% (75/75; 95.1–100.0%)	0.0% (0/75; 0.0–3.9%)
	**FluA**	100.0% (15/15; 79.6–100.0%)	100.0% (60/60; 94.0–100.0%)	100.0% (75/75; 95.1–100.0%)	0.0% (0/75; 0.0–3.9%)
	**FluB**	100.0% (15/15; 79.6–100.0%)	100.0% (60/60; 94.0–100.0%)	100.0% (75/75; 95.1–100.0%)	0.0% (0/75; 0.0–3.9%)
	**RSV**	100.0% (15/15; 79.6–100.0%)	100.0% (60/60; 94.0–100.0%)	100.0% (75/75; 95.1–100.0%)	0.0% (0/75; 0.0–3.9%)
**Xpert Xpress CoV-2/Flu/RSV plus**	**SARS-CoV-2**	100.0% (15/15; 79.6–100.0%)	100.0% (60/60; 94.0–100.0%)	100.0% (75/75; 95.1–100.0%)	0.0% (0/75; 0.0–3.9%)
	**FluA**	100.0% (15/15; 79.6–100.0%)	100.0% (60/60; 94.0–100.0%)	100.0% (75/75; 95.1–100.0%)	0.0% (0/75; 0.0–3.9%)
	**FluB**	100.0% (15/15; 79.6–100.0%)	100.0% (60/60; 94.0–100.0%)	100.0% (75/75; 95.1–100.0%)	0.0% (0/75; 0.0–3.9%)
	**RSV**	100.0% (15/15; 79.6–100.0%)	100.0% (60/60; 94.0–100.0%)	100.0% (75/75; 95.1–100.0%)	0.0% (0/75; 0.0–3.9%)
**Panther Fusion SARS-CoV-2/Flu A/B/RSV Assay**	**SARS-CoV-2**	100.0% (15/15; 79.6–100.0%)	100.0% (60/60; 94.0–100.0%)	100.0% (75/75; 95.1–100.0%)	0.0% (0/75; 0.0–3.9%)
	**FluA**	100.0% (15/15; 79.6–100.0%)	100.0% (60/60; 94.0–100.0%)	100.0% (75/75; 95.1–100.0%)	0.0% (0/75; 0.0–3.9%)
	**FluB**	100.0% (15/15; 79.6–100.0%)	100.0% (60/60; 94.0–100.0%)	100.0% (75/75; 95.1–100.0%)	0.0% (0/75; 0.0–3.9%)
	**RSV**	100.0% (15/15; 79.6–100.0%)	100.0% (60/60; 94.0–100.0%)	100.0% (75/75; 95.1–100.0%)	0.0% (0/75; 0.0–3.9%)

Note: For each target, positive % agreement was calculated using the 15 positive specimens available for that pathogen (*n* = 15), whereas negative % agreement was calculated using the remaining 60 specimens, comprising 45 specimens positive for other respiratory pathogens but confirmed negative for the target under evaluation and 15 specimens confirmed negative for all evaluated pathogens. Overall % agreement and discordance % were calculated using all 75 specimens included in the principal viral analysis.

**Table 2:** Summary of paired statistical comparisons of Ct values between UBM and comparator media across molecular platforms and targets. The table reports the results of paired statistical analyses performed to compare cycle threshold (Ct) values obtained from samples processed in Universal Biomolecular Medium (UBM) versus comparator media (UTM or Panther Fusion Specimen Lysis Tubes buffer, depending on platform). Results are stratified by viral target and molecular assay. For each comparison, the test statistic and corresponding *p* value are reported. Statistically significant differences (*p* < 0.05) are indicated with an asterisk (*).

Target	Allplex SARS-CoV-2/FluA/FluB/RSV Assay			Xpert Xpress CoV-2/Flu/RSV Plus		Panther Fusion SARS-CoV-2/Flu A/B/RSV Assay
**SARS-CoV-2**	**N gene**		Paired-t test t(14) = 5.8, *p* < 0.001 *	Paired-t test t(14) = 0.5, *p* = 0.626		Paired-t test t(14) = 2.3, *p* = 0.040 *
	**S gene**		Paired-t test t(14) = 1.5, *p* = 0.163			
	**RdRp gene**		Paired-t test t(14) = 3.5, *p* = 0.004 *			
**FluA**	Paired-t test t(14) = 2.2, *p* = 0.043 *			**FluA1**	Paired Wilcoxon test Z = −0.4, *p* = 0.607	Paired Wilcoxon test Z = −0.5, *p* = 0.589
				**FluA2**	Paired Wilcoxon test Z = −0.6, *p* = 0.532	
**FluB**	Paired-t test t(14) = 0.1, *p* = 0.906			Paired-t test t(14) = 2.9, *p* = 0.012 *		Paired-t test t(14) = 0.1, *p* = 0.900
**RSV**	Paired Wilcoxon test W+ = 85, *p* = 0.164			Paired-t test t(14) = 0.7, *p* = 0.492		Paired-t test t(14) = 2.9, *p* = 0.011 *
**Negative**	**Exo IC**	Paired-t test t(14) = 0.09, *p* = 0.931		Paired-t test t(14) = 1.1, *p* = 0.301		Paired-t test t(14) = 2.2, *p* = 0.041 *
	**Endo IC**	Paired-t test t(14) = 0.1, *p* = 0.886				

**Table 3:** Bland–Altman estimates for paired Ct differences between UBM and the corresponding comparator condition. Mean bias and limits of agreement are reported with their 95% confidence intervals.

Assay	Target	Mean bias (95% CI), Ct	Lower LoA (95% CI), Ct	Upper LoA (95% CI), Ct
**Allplex SARS-CoV-2/FluA/FluB/RSV Assay**	**SARS-CoV-2 S**	0.73 (−0.33 to 1.80)	−3.05 (−4.92 to −1.18)	4.52 (2.65 to 6.39)
	**SARS-CoV-2 RdRp**	−2.10 (−3.39 to −0.81)	−6.66 (−8.91 to −4.41)	2.46 (0.21 to 4.71)
	**SARS-CoV-2 N**	−3.45 (−4.72 to −2.18)	−7.95 (−10.18 to −5.73)	1.05 (−1.17 to 3.28)
	**FluA**	−0.79 (−1.54 to −0.03)	−3.47 (−4.79 to −2.14)	1.90 (0.57 to 3.22)
	**FluB**	−0.02 (−0.46 to 0.41)	−1.58 (−2.35 to −0.81)	1.53 (0.76 to 2.30)
	**RSV**	0.35 (−1.09 to 1.80)	−4.76 (−7.29 to −2.23)	5.47 (2.94 to 8.00)
	**Endogenous IC**	−0.06 (−0.99 to 0.87)	−3.35 (−4.97 to −1.73)	3.22 (1.60 to 4.85)
	**Exogenous IC**	0.01 (−0.25 to 0.27)	−0.91 (−1.37 to −0.46)	0.93 (0.48 to 1.39)
**Xpert Xpress CoV-2/Flu/RSV Plus**	**SARS-CoV-2**	0.11 (−0.35 to 0.57)	−1.52 (−2.32 to −0.72)	1.73 (0.93 to 2.53)
	**FluA1**	−0.37 (−1.08 to 0.34)	−2.89 (−4.13 to −1.65)	2.14 (0.90 to 3.38)
	**FluA2**	−0.41 (−1.13 to 0.30)	−2.95 (−4.20 to −1.70)	2.12 (0.87 to 3.38)
	**FluB**	0.52 (0.13 to 0.91)	−0.85 (−1.52 to −0.17)	1.89 (1.21 to 2.56)
	**RSV**	0.25 (−0.52 to 1.02)	−2.47 (−3.82 to −1.13)	2.98 (1.63 to 4.33)
	**IC**	−0.12 (−0.36 to 0.12)	−0.97 (−1.39 to −0.55)	0.73 (0.31 to 1.15)
**Panther Fusion SARS-CoV-2/Flu A/B/RSV Assay**	**SARS-CoV-2**	−0.31 (−0.60 to −0.02)	−1.34 (−1.85 to −0.83)	0.72 (0.22 to 1.23)
	**FluA**	−0.02 (−0.46 to 0.42)	−1.59 (−2.37 to −0.82)	1.55 (0.78 to 2.33)
	**FluB**	−0.05 (−0.83 to 0.73)	−2.80 (−4.17 to −1.44)	2.71 (1.35 to 4.07)
	**RSV**	−1.09 (−1.89 to −0.29)	−3.93 (−5.33 to −2.53)	1.74 (0.34 to 3.14)
	**IC**	0.70 (0.03 to 1.37)	−1.66 (−2.83 to −0.50)	3.06 (1.90 to 4.23)

**Table 4:** Analytical performance of UBM compared with eSwab for the detection of hMPV, HRV, *H. influenzae*, *M. pneumoniae* and *B. pertussis*. Results are reported for each tested assay and each target organism. Positive % agreement, negative % agreement, overall % agreement and discordance % are reported for each target organism. Numerators and denominators (*n*/*N*) indicate the number of concordant results over the total number of expected positive or negative samples. Ninety-five percent confidence intervals (95% CI) are shown in brackets. Confidence intervals for proportions were calculated using the Wilson score method. Percentages are reported using the decimal separator applied throughout the manuscript.

Target	Positive % Agreement [*n*/*N*; 95% CI]	Negative % Agreement [*n*/*N*; 95% CI]	Overall % Agreement [*n*/*N*; 95% CI]	Discordance % [*n*/*N*; 95% CI]
**hMPV**	100.0% (5/5; 56.6–100.0%)	100.0% (25/25; 86.7–100.0%)	100.0% (30/30; 88.6–100.0%)	0.0% (0/30; 0.0–9.5%)
**HRV**	100.0% (5/5; 56.6–100.0%)	100.0% (25/25; 86.7–100.0%)	100.0% (30/30; 88.6–100.0%)	0.0% (0/30; 0.0–9.5%)
***H. influenzae***	100.0% (5/5; 56.6–100.0%)	100.0% (25/25; 86.7–100.0%)	100.0% (30/30; 88.6–100.0%)	0.0% (0/30; 0.0–9.5%)
***M. pneumoniae***	100.0% (5/5; 56.6–100.0%)	100.0% (25/25; 86.7–100.0%)	100.0% (30/30; 88.6–100.0%)	0.0% (0/30; 0.0–9.5%)
***B. pertussis***	100.0% (5/5; 56.6–100.0%)	100.0% (25/25; 86.7–100.0%)	100.0% (30/30; 88.6–100.0%)	0.0% (0/30; 0.0–9.5%)

Note: For each target, positive % agreement was calculated using the 15 positive specimens available for that pathogen (*n* = 15), whereas negative % agreement was calculated using the remaining 60 specimens, comprising 45 specimens positive for other respiratory pathogens but confirmed negative for the target under evaluation and 15 specimens confirmed negative for all evaluated pathogens. Overall % agreement and discordance % were calculated using all 75 specimens included in the pilot viral analysis.

## Data Availability

The original contributions presented in this study are included in the article. Further inquiries can be directed to the corresponding author.

## Abbreviations

The following abbreviations are used in this manuscript:

UBM™	Universal Biomolecular Medium
UTM^®^	Universal Transport Medium
RT-PCR	Real-Time Polymerase Chain Reaction
PCR	Polymerase Chain Reaction
Ct	Cycle Threshold
SARS-CoV-2	Severe Acute Respiratory Syndrome Coronavirus 2
FluA	Influenza A Virus
FluB	Influenza B Virus
RSV	Respiratory Syncytial Virus
HRV	Human Rhinovirus
hMPV	Human Metapneumovirus
MPV	Human Metapneumovirus (assay designation)
*H. influenzae*	*Haemophilus influenzae*
*M. pneumoniae*	*Mycoplasma pneumoniae*
*B. pertussis*	*Bordetella pertussis*
CI	Confidence Interval
ERVISS	European Respiratory Virus Surveillance Summary
GISRS	Global Influenza Surveillance and Response System
EU/EEA	European Union/European Economic Area
RdRp	RNA-dependent RNA Polymerase
ORF1ab	Open Reading Frame 1ab
N gene	Nucleocapsid Gene
S gene	Spike Gene
E gene	Envelope Gene
M gene	Matrix Gene
NS gene	Non-Structural Gene
NS2 gene	Non-Structural Protein 2 Gene
PB2	Polymerase Basic Protein 2
PA	Polymerase Acidic Protein
IC	Internal Control
Exo IC	Exogenous Internal Control
Endo IC	Endogenous Internal Control
IFU	Instructions for Use
GDPR	General Data Protection Regulation
